# Does intranasal naloxone administration increase the risk of 2019 coronavirus disease transmission?

**DOI:** 10.1017/cem.2020.395

**Published:** 2020-05-12

**Authors:** Yuen Chin Leong, P. Richard Verbeek

**Affiliations:** * Sunnybrook Centre for Prehospital Medicine, Sunnybrook Health Sciences Centre, Toronto, ON; † Faculty of Medicine, University of Toronto, Toronto, ON; ‡ Division of Emergency Medicine, Department of Medicine, University of Toronto, Toronto, ON

**Keywords:** COVID-19, emergency medicine, prehospital/EMS, substance misuse

Dear Editor,

On April 3, 2020, several news outlets reported that North Bay firefighters were to stop using naloxone nasal spray as a result of the 2019 coronavirus disease (COVID-19) pandemic. This has raised concerns whether intranasal naloxone is an aerosol generating procedure (AGP) that may increase the risk of COVID-19 transmission.^1,2^ Despite an extensive online search, we have been unable to identify any published materials that address this issue.

Naloxone is usually administered to opioid overdose patients who experience severe respiratory depression.^3^ Intranasal naloxone is administered by placing the patient supine, tilting the head into a sniffing position, inserting the nozzle of the device into the nostril, and pressing the plunger to release the drug. In this context, commercially available Narcan^TM^ Nasal Spray produces particles where 90% are > 60 microns in size,^4^ whereas many Ontario paramedic services use the Intranasal Mucosal Atomization Device (MAD),^5^ which produces particles ranging from 30–100 microns. Due to the large particle size (> 20 microns),^1,2^ each device will result in drug deposition in the nasopharyngeal cavity even if the patient inhales at the time the plunger is pressed.

Accordingly, administration of intranasal naloxone should not be considered an AGP. However, because MAD requires a higher volume of solution (1 mg/ml) compared to Narcan^TM^ Nasal Spray (4 mg/0.1ml), we have advised paramedics to use alternative effective routes of naloxone administration (e.g., intramuscular or intravenous). Following intranasal naloxone, it is advisable to apply a surgical mask to patients who are emerging into consciousness because it has been shown that, in a simulated sneeze event,^2^ aerosol generation can extend up to 66 cm but is eliminated by a surgical mask.

It would also be prudent for all prehospital care providers to consider wearing an appropriate protective mask according to public health guidance and then to exercise physical distancing once the naloxone has been administered. Whatever the route, we also advocate that paramedics and firefighters administer naloxone as their initial step with a goal of avoiding bag-valve-mask (BVM) ventilation where appropriate because this is well known to be a high-risk AGP. The decision to assist with BVM ventilation is a difficult choice and should be done with an abundance of caution using a tight two-handed seal with minimal tidal volumes.

In conclusion, we support the administration of commercially available naloxone nasal spray as a non-AGP. It should not be withheld from patients experiencing an opioid overdose.
